# Eye spy a liar: assessing the utility of eye fixations and confidence judgments for detecting concealed recognition of faces, scenes and objects

**DOI:** 10.1186/s41235-020-00227-4

**Published:** 2020-08-14

**Authors:** Ailsa E. Millen, Lorraine Hope, Anne P. Hillstrom

**Affiliations:** Department of Psychology, University of Portsmouth, Portsmouth, England, UK

**Keywords:** Concealed recognition, Familiarity, Concealed knowledge, Eye fixations, Confidence, Meta-cognition, Deception

## Abstract

**Background:**

In criminal investigations, uncooperative witnesses might deny knowing a perpetrator, the location of a murder scene or knowledge of a weapon. We sought to identify markers of recognition in eye fixations and confidence judgments whilst participants told the truth and lied about recognising faces (Experiment 1) and scenes and objects (Experiment 2) that varied in familiarity. To detect recognition we calculated effect size differences in markers of recognition between familiar and unfamiliar items that varied in familiarity (personally familiar, newly learned).

**Results:**

In Experiment 1, recognition of personally familiar faces was reliably detected across multiple fixation markers (e.g. fewer fixations, fewer interest areas viewed, fewer return fixations) during honest and concealed recognition. In Experiment 2, recognition of personally familiar non-face items (scenes and objects) was detected solely by fewer fixations during honest and concealed recognition; differences in other fixation measures were not consistent. In both experiments, fewer fixations exposed concealed recognition of newly learned faces, scenes and objects, but the same pattern was not observed during honest recognition. Confidence ratings were higher for recognition of personally familiar faces than for unfamiliar faces.

**Conclusions:**

Robust memories of personally familiar faces were detected in patterns of fixations and confidence ratings, irrespective of task demands required to conceal recognition. Crucially, we demonstrate that newly learned faces should not be used as a proxy for real-world familiarity, and that conclusions should not be generalised across different types of familiarity or stimulus class.

## Statement of significance

Detecting recognition of faces, scenes and objects related to a crime using visual evidence is critical to the investigative process. But witnesses sometimes lie about their knowledge, whether to conceal involvement in the crime or due to fear of retribution. In such cases, unobtrusive methods to index recognition may be useful for uncovering concealed knowledge.

Tracking eye movements during inspection of photographs offers one approach to detect concealed recognition, but findings to date vary substantially depending on a complex interplay between stimulus class (e.g. faces vs non-face items) and degree of familiarity (e.g. personally familiar vs newly learned). We assessed the utility of eye fixations and confidence judgments to detect recognition of faces, scenes and objects that vary in familiarity.

Markers of recognition for personally familiar faces were robust irrespective of honesty. Markers of recognition for newly learned faces differed across honest and concealed trials and did not exhibit the same consistent differences observed for personally familiar faces. Our findings suggest that newly learned faces are processed similarly to unfamiliar faces and that this limits the scope for recognition detection. Recognition of personally familiar and newly learned non-face items was solely differentiated by fewer fixations, but there were no reliable differences in the spatial distribution of fixations. Differences in confidence judgments reliably indicated recognition for personally familiar faces and honest responses to personally familiar non-face items. The varying effects of stimulus class, degree of familiarity and task instructions on fixation patterns pose a challenge for recognition detection by eye fixations.

## Introduction

Concealed knowledge tests employed in field practice typically use autonomic measures such as skin conductance to detect crime details (Ben-Shakhar & Elaad, 2003; Gamer, 2011; Gamer, Verschuere, Crombez, & Vossel, 2008; Meijer, Klein Selle, Elber, & Ben-Shakhar, 2014). However, less obtrusive methods are desirable for application in a wider range of security settings, and remote eye tracking technology shows distinct promise for field application. Several recent experiments demonstrated the efficacy of eye fixation variables for the detection of concealed knowledge (Lancry-Dayan, Nahari, Ben-Shakhar, & Pertzov, 2018; Mahoney, Kapur, Osmon, & Hannula, 2018; Millen, Hope, Hillstrom, & Vrij, 2017; Peth, Kim, & Gamer, 2013; Peth, Suchotzki, & Gamer, 2016; Schwedes & Wentura, 2012; Schwedes & Wentura, 2016; for a review see Gamer & Pertzov, 2018). Some research suggests that eye tracking tests can outperform traditional autonomic measures of concealed knowledge (e.g. Lancry-Dayan et al., 2018), whereas others present more cautious conclusions (Peth et al., 2013). Variability in detection rates across experiments may be partly explained by the different stimuli depicted across studies (e.g. faces, scenes or more general objects) and the strength of familiarity of those people, places and objects at recognition.

## Using fixations to detect recognition

Emergent research suggests that recognition detection of personally familiar faces is robust, such that fixation patterns for personally familiar faces are easily distinguished from genuinely unfamiliar faces despite cognitive effort to conceal recognition during explicit denial (Millen et al., 2017) and informed strategies to beat the test (herein referred to as countermeasures; Lancry-Dayan et al., 2018; Millen & Hancock, 2019, this issue). Millen et al. (2017, 2019) and Lancry-Dayan et al. (2018) found large and consistent effect sizes for the detection of personal familiarity despite using different display formats (sequential vs simultaneous respectively) and procedural methods. Findings for less familiar faces (e.g. one brief exposure and famous celebrities) are smaller and less robust (Millen et al., 2017). These findings are in line with existing research indicating that exposure to variability in appearance and personal social and emotional experiences (i.e. personal familiarity) facilitates fast, reflexive recognition (Gobbini & Haxby, 2007; Natu & Toole, 2011; Wiese et al., 2019). Beyond faces, these findings are consistent with Peth et al. (2013), who reported less robust findings for incidentally acquired knowledge of objects during a mock crime; items central to the crime were successfully detected, but those peripheral to it were not. To our knowledge, no published research has systematically explored the robustness of fixation-based recognition markers of personally familiar photographs across face and non-face stimuli or compared the relative ease of detecting personal familiarity over newly learned items. Newly learned items are commonly used in tests of concealed knowledge detection.

## Predicting patterns of fixations based on recognition memory

Differences in patterns of fixations during recognition are well established in the face recognition literature. Accurate familiar face identification is reported within two fixations (Hsiao & Cottrell, 2008; for a review see Hannula et al., 2010), which is consistent with theoretical models of recognition positing that truly familiar faces are recognised faster and more accurately than relatively unfamiliar faces (Bruce & Young, 1986; Burton, Bruce, & Hancock, 1999; Burton, Jenkins, & Schweinberger, 2011; Gobbini et al., 2013; Gobbini & Haxby, 2007; Hancock, Bruce, & Burton, 2000; Johnston & Edmonds, 2009; Schacter, Norman, & Koutstaal, 1998). Fast recognition of a familiar face requires fewer fixations, less distributed viewing patterns (e.g. across the eyes, nose and mouth), proportionately less viewing of inner face regions and fewer return fixations to regions of the face previously viewed, than for unknown faces (e.g. Althoff & Cohen, 1999). This overall reduction in visual sampling for familiar faces is also reflected in average fixation durations, which tend to be longer for familiar faces (e.g. Ryan, Hannula, & Cohen, 2007). Fewer fixations of longer durations reflect less cognitive effort during recognition of faces we know—a consequence of robust memory representations (Balas, Cox, & Conwell, 2007; Natu & Toole, 2011; Ramon & Gobbini, 2017). Conversely, systematic viewing patterns and shorter fixation durations for unfamiliar face identification reflect optimal but effortful information extraction for accurate rejection. This reduction in visual exploration for familiar faces is thought to represent a shift from part-based to holistic processing (Bobak, Parris, Gregory, Bennetts, & Bate, 2017; Collishaw & Hole, 2000; Farah, Wilson, Drain, & Tanaka, 1998; Richler, Mack, Gauthier, & Palmeri, 2009), which occurs as an unfamiliar face becomes familiar via repeated exposures. Nevertheless, Althoff (1998; see also Althoff et al., 1999) originally claimed that the effects of recognition on eye fixations are observable after three exposures to a photograph of a face. While we know that pixel-to-pixel memory is not the same as familiar face recognition proper (Burton, Bruce & Hancock, 1999; Ritchie & Burton, 2017), the impact of substituting personal familiarity with newly learned images is unknown in the context of detecting concealed knowledge by eye tracking.

Fewer fixations are also reported during recognition of non-face photographs such as buildings (Althoff & Cohen, 1999) and scenes (e.g. Johnston & Leek, 2009; Kafkas & Montaldi, 2012; Leek et al., 2012; Ryan et al., 2007; Ryan, Althoff, Whitlow, & Cohen, 2000). However, where and how people look at non-face photographs such as complex scenes or general objects is arguably more variable than it is for faces, partly because these images are less homogeneous. Here, we evaluate a range of eye fixation parameters to assess the reliability of different variables for detecting recognition across varying degrees of familiarity (personally familiar and newly learned) and different stimuli classes (faces and non-face items).

In the eye movement analyses we set out to achieve three main objectives. (1) To further test the robustness of differences in patterns of fixations for the detection of personally familiar faces (e.g. Millen et al., 2017). Here we explicitly instructed participants to adopt spontaneous strategies to beat the test. We motivated deceivers with a cash incentive if they managed to evade detection. In Millen et al.’s (2017) original study, only 32% attempted to conceal recognition using deliberate countermeasure strategies, meaning that the robustness of their fixation markers may have been overestimated. (2) To assess whether the markers of recognition for personally familiar faces in Experiment 1 are the same as markers of recognition for personally familiar non-face items such as places and objects in Experiment 2. (3) Within each experiment, we compared results for personally familiar items with new images that were learned to criterion in a study phase (100% face-name pairings) before lying about the same images at test. Furthermore, in an entirely novel strand of the research, we examine whether patterns of verbal confidence judgments differ for familiar and unfamiliar items and how these patterns change across stimulus class, familiarity and explicit denial of knowledge.

## Predicting patterns of confidence based on recognition memory

In the legal system, reports of high confidence are known to impair the ability to detect deception (Tetterton, 2005). Crucially, professionals are no better than lay persons at distinguishing the veracity of confidence-accuracy relationships (Bond & DePaulo, 2016; Vrij, Fisher, Mann, & Leal, 2008; DePaulo, Charlton, Cooper, Lindsay, & Muhlenbruck, 1997). Our rationale for studying confidence is based on two main theoretical frameworks: First, models of recognition support that recall of well-encoded memories is typically fast and accompanied by high ratings of subjective confidence about those decisions (Ellis, Shepherd, & Davies, 1979; Lefebvre, Marchand, Smith, & Connolly, 2009; Sauerland, Sagana, Sporer, & Wixted, 2018; Weber & Brewer, 2006; Wells & Olson, 2003; Wixted & Wells, 2017). Second, eye movements during decision making reflect deliberations and decision outcomes (Chua, Hannula, & Ranganath, 2012; Glaholt & Reingold, 2011; Russo, 2011). Assuming confidence judgments for familiar items are more stable, confidence judgments should be higher and less variable during recognition than genuine deliberations during correct rejection of genuinely unfamiliar items. The lack of genuine deliberation during recognition should also be reflected in reduced fixation patterns (fewer fixations, fewer areas of scale viewed, fewer return fixations to the scale) and longer fixation durations during viewing of the confidence scale. Our specific predictions are that confidence judgments for genuine responses to learned or personally known items will be higher and more homogeneous (less variable) than those to genuinely unfamiliar items. In addition, liars might also exploit the belief that high confidence reports are convincing by providing well-rehearsed, consistent and confident memory reports when questioned (i.e. increased homogeneity in verbal reports).

## Method

### Power

In both experiments, we aimed for a minimum of 24 participants. The planned sample size was based on a G*Power analysis (Faul, Erdfelder, Lang, & Buchner, 2007) with *d* = 0.7, an α error probability of .05 and a β of .95, which estimated 24 participants. The predicted effect size for the current study is conservative compared to a similar study by Millen et al. (2017), who reported large effect sizes for detection of personally familiar faces by number of fixations (*d* = 0.9), number of areas of the face viewed (*d* = 0.9) and average fixation duration (*d* = 0.9). Since we did not have a script to directly determine exclusions at the point of collecting these data, the total number of valid participants for analysis exceeded the minimum required sample size.

### General design

In both experiments, participants viewed colour photographs of familiar and unfamiliar faces (Experiment 1) or familiar and unfamiliar non-face items (objects and scenes; Experiment 2). In each experiment participants first completed one practice block during which they told the truth about all items, followed by two concealed recognition blocks. In the concealed blocks, participants denied recognition of one familiar face type (newly learned or personally familiar) whilst telling the truth about all other faces. These three basic task instructions, deny recognition of one set of familiar ‘probes’; correctly reject unfamiliar ‘irrelevant’ items; and honestly respond to a different set of familiar ‘targets’, are similar to the Concealed Information Test (CIT) three-stimulus protocol (e.g. Sauerland, Wolfs, Crans, & Verschuere, 2017; Seymour & Kerlin, 2008; Verschuere, Crombez, Degrootte, & Rosseel, 2010). Each different type of familiarity (e.g. unfamiliar, newly learned, personally familiar) was presented in equal trial numbers such that multiple probes and targets were presented in each block. Participants made yes/no responses via a button press whilst verbally stating their response out loud at the same time. The buttons assigned to yes/no responses were counterbalanced by hand dominance.

In Experiment 1, participants viewed 40 faces per block: 10 unfamiliar, 10 newly learned, 10 personally familiar and 10 famous celebrities. In one concealed block, participants denied recognition of newly learned ‘probe’ faces, whilst correctly rejecting unfamiliar ‘irrelevant’ faces and honestly identifying personally familiar ‘target’ faces and famous ‘target’ faces. In the other concealed block, participants denied recognition of personally familiar ‘probe’ faces, whilst correctly rejecting unfamiliar ‘irrelevant’ faces and honestly identifying newly learned ‘target’ faces and celebrity ‘target’ faces. The 10 celebrity faces were included to replicate the design of Millen et al. (2017). In the current experiment we were not interested in concealed knowledge of famous celebrities; thus, for the purpose of the current study, these were filler items and were not analysed further. The set of personally familiar faces for each group of participants (10 participant tutees in each group) included the participant’s own face. In the ‘deny personally familiar faces’ block, participants were instructed to also deny recognition of their own face (excluded from analyses). In the ‘deny recognition of newly learned faces’ block, participants were instructed to honestly identify their own face (excluded from analyses) whilst also honestly identifying the other personally familiar faces.

In Experiment 2, the design was the same except that participants were not presented with equivalent famous non-face items. Accordingly, participants viewed 30 non-face items in each block: 10 unfamiliar, 10 newly learned, 10 personally familiar. In one concealed block, participants denied recognition of newly learned non-face probe items, whilst correctly rejecting unfamiliar non-face ‘irrelevant’ items and honestly identifying personally familiar non-face items. In the other concealed block, participants denied recognition of personally familiar non-face probe items, whilst correctly rejecting unfamiliar ‘irrelevant’ non-face items and honestly identifying newly learned ‘target’ non-face items.

### Participants

Thirty-three undergraduate students (26 females, 7 males) participated in Experiment 1 (range 19–21 years, *M* = 20.3, *SD* = 1.9), and 38 undergraduate students (36 females, 2 males) participated in Experiment 2 (range 19–27 years, *M* = 20.31, *SD* = 1.59). All participants had normal or corrected-to-normal vision. All participants received £5 remuneration for taking part.

### Apparatus and materials

Participants’ eye movements were tracked using the Eyelink II Head Mounted Eye Tracker (SR Research, Ottawa, ON, Canada) at a recording rate of 250 Hz. Fixations were defined by Eyelink’s online standard parser configuration as an eye event that was not a blink or saccade. Using the standard cognitive configuration, saccades were defined as eye movements that exceeded 30°/s velocity or 8000°/s^2^ acceleration (SR Research Eyelink II User Manual, Version 2.14). We additionally set a fixation threshold of 100 ms. The system has a spatial resolution of less than 0.01° and a spatial accuracy of 0.5°. Manual button press responses were collected by a Microsoft Sidewinder Plug-and-Play game pad and relayed back to the host computer.

In Experiment 1, a total of 120 face photographs were presented over the practice and two concealed recognition trial blocks (10 unfamiliar identities, 10 newly learned items, 10 personally known items and 10 famous faces per block). In Experiment 2, a total of 90 (10 unfamiliar identities, 10 newly learned items, 10 personally known items) non-face photographs were presented across the practice and two concealed recognition blocks. Photographs were presented using Experiment Builder (Version 1.6.121, SR Research) on a desktop computer linked to a 19-in. CRT monitor (model G90FB, resolution 1280 × 1024 pixels, refresh rate 89 Hz). Images were presented randomly to the left (292, 292) or the right side (704, 292) of the screen to minimise anticipatory guessing behaviour of picture location.

#### Faces

All photographs were portraits showing the full face of a person with a neutral expression and eye gaze towards the camera. The appearance of all photographs was standardised using Adobe Photoshop Elements (Versions 2.0, CS4) for the removal of red-eye, accessories and jewellery, and they were extracted from their original background to a blue background (HEX: #377BE8) measuring (640 × 480 pixels). Unfamiliar faces were sourced with permission from academic databases (Burton, White, & McNeill, 2010; Psychological Image Collection at Stirling (PICS), http://pics.stir.ac.uk (Hancock, n.d.); Weyrauch, Heisele, Huang, & Blanz, 2004) and individuals from schools and universities (Taunton College, University of Stirling) who volunteered to have their photographs taken to create an unfamiliar face database for the purposes of the present experiment. Newly learned faces were sourced from our unfamiliar face database and familiarised using a learn-to-criterion procedure (Schyns, Bonnar, & Gosselin, 2002) directly before performing the concealed knowledge test. Personally familiar faces were photographs of the student participants. Participants were recruited from pre-existing tutorial groups (established at the start of term by random allocation to class lists) to ensure a baseline of real-world familiarity. There were 10 participants in each group. At the time of the experiment, participants were personally acquainted with fellow tutees for approximately 5 months. Multiple images were taken of each person (all with gaze facing forward) so that a unique image of that person could be used in each trial block. Personally familiar faces were matched to newly learned and unfamiliar faces matched by hair colour, eye colour and skin tone. For newly learned and unfamiliar faces, new sets of images were used for each trial block but, within blocks, the same newly learned image was presented at study and test, since we specifically wanted to investigate whether using such familiarisation procedures (e.g. Seymour, Baker, & Gaunt, 2013) produced results equivalent to personal familiarity.

#### Non-face items (scenes and objects)

Photographs of personally familiar objects and scenes were specific to psychology student participants at the university. The photographs comprised colour images of the city guildhall, entrance to psychology department, entrance to student union, entrance to university library, coursework submission point, university logo, log-in to electronic resources webpage, coursework submission cover sheet, university hoodie and student ID card. The first five items represented scenes, whereas the last five more generally represented objects, all of which were recognisable to participants. For consistency, the photographs for irrelevant and newly learned categories followed the same structure (e.g. photographs of libraries and sweatshirts from other universities). All images were taken with a SONY Cyber-shot camera specifically for the experiment. Photographs were re-sized to 640 × 480 pixels and presented on a blue background (HEX: #5DBCD2) using Adobe Photoshop CS5.1.

*Note*: The same personally familiar items (faces, scenes and objects) were presented in both blocks of trials but using different photographic images. Newly learned and unfamiliar items were unique to each block.

### Procedure

Participants were seated in a controlled, quiet and dimly lit room 0.80 m from the display screen. Light levels were carefully controlled for all participants via a dimmer switch. The Eyelink II headband was comfortably secured to the participant’s head.

#### Rate personally familiar items

Prior to the test, participants were presented with photographs of personally familiar items on a paper hand-out. Participants were asked to look at each photograph in turn and to indicate, yes or no, if they recognised the face, scene or object in the photograph. These images were not the same as the ones presented in the experimental trials. The participant rated each photograph for familiarity (1 = not at all familiar, 7 = most familiar) and noted down the name of the face, scene or object. Ratings taken prior to the experiment confirmed personally known faces (*M* = 4.90, *SD* = 0.82) and personally familiar non-face items (*M* = 6.59, *SD* = 1.05) as familiar.

#### Study phase

Prior to the CIT, participants were shown 10 photographs of faces (Experiment 1) or a selection of scenes and objects (Experiment 2) and asked to learn them for the task. Unfamiliar items were presented on 10 individual colour photograph cards (6 × 8 cm). These were laid out on the table in front of the participant, who was asked to study them until he/she perceived them as learned. A name was then placed below each photograph, and participants were then instructed to learn which name belonged to each photograph (e.g. ’Jack Wilson’ or ’Grantham Guildhall’). Once the participant indicated that he/she had learned the item-name pairings, the cards were shuffled and handed back to the participant. The participant matched each photograph to its name tag correctly. The criterion for learning was that participants pair all photograph-name pairings correctly 100% twice consecutively (procedure based on Schyns et al., 2002). There was no time limit for the successful completion of the study phase. Upon completion, measurement of the participant’s eye movements was calibrated for accurate eye movement monitoring by the eye tracker.

#### Calibration

Eye position was calibrated using a 3 × 3 dot array prior to each test block and, when necessary, in between condition blocks. To calibrate, a black dot with a white centre was presented in the middle of the screen. The participant fixated the white centre of the black dot, and the experimenter accepted the fixation by pressing a button on the host computer. Once the initial fixation was accepted, the same dot was displayed randomly, one location at a time, using a 3 × 3 array. The host computer was operated by the experimenter and was positioned behind the participant and the display computer. Following calibration, participants were informed that eye movements were carefully monitored by the experimenter, from a separate host computer behind them, with the explicit purpose of detecting concealed recognition. They were advised that the experimenter would record eye movement data such as where they looked on the screen and for how long, and how often they looked away and looked back. They were not advised which eye movements might indicate deception. The purpose here was to evaluate if participants would or could control their eye movements to conceal recognition spontaneously. The emphasis on the monitoring of eye movements aimed to increase processes of monitoring and control and the employment of countermeasure strategies.

#### Test phase

**Fig. 1:**
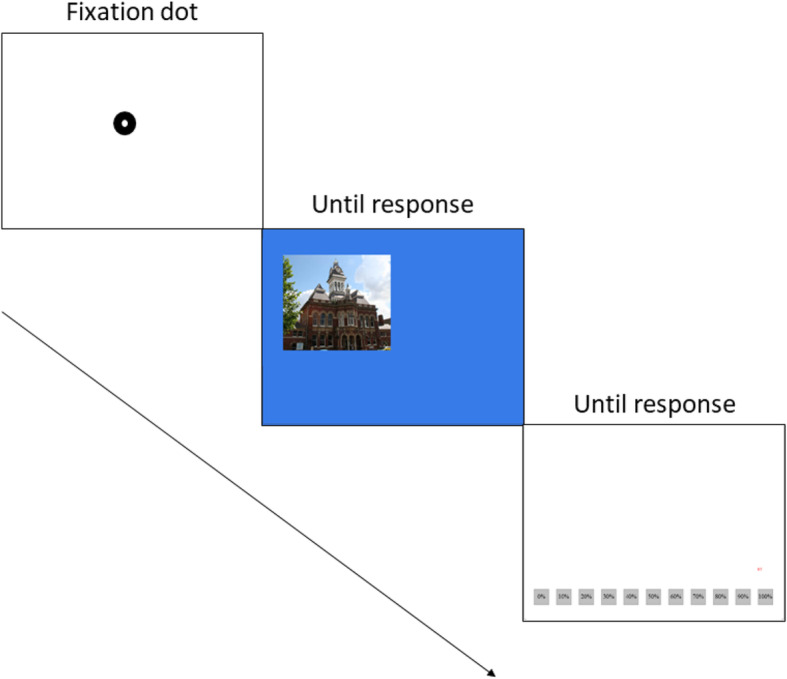
An example of one full trial sequence. The trial commenced with a drift correction followed by a photograph for recognition (the picture depicted here is a personally familiar place, the city guildhall), followed by a confidence scale (0–100%). In one block, the participant was instructed to conceal knowledge of the personally familiar place by pressing a button assigned to the ‘unfamiliar’ response whilst concurrently saying ‘unfamiliar’ out loud. In the same block, following a similar procedure, participants honestly identified photographs of newly learned items (pressed and said ‘familiar’) and correctly rejected unfamiliar items (pressed and said ‘unfamiliar’) . In the other block, the participant was instructed to honestly identify this personally familiar place by pressing a button assigned to the ‘familiar’ response whilst concurrently saying ‘familiar’ out loud. In that block, participants denied recognition of newly learned items and correctly rejected unfamiliar items. Following the recognition judgment, participants were asked to report how confident they were in their recognition judgment. The instruction for recognition and confidence judgments was that they should try to appear honest even when they were lying. Photographs for recognition were presented randomly to the left (292, 292) or the right side (704, 292) of the drift correction dot. Confidence scales were presented randomly at either the top or the bottom of the screen

See Fig. 1 for an example of a full trial sequence. Participants were presented with a fully randomised sequence of familiar and unfamiliar colour photographs. Prior to the display of each face, a fixation dot was shown in the centre of the screen to correct any drift in eye movements following the initial calibration. Participants responded by making a dichotomous ‘familiar’ or ‘unfamiliar’ button press response whilst at the same time verbalising their response. Directly after the button press was made, the photograph was removed from the screen and a confidence scale was presented. Participants were instructed to look at the scale (0–100%) and decide how confident they were about their previous familiarity judgment. Participants indicated their confidence judgment by pressing a trigger button on the game pad (any) whilst verbalising their rating (numerators of 10, i.e. ’ninety’). There was no upper time limit for recognition or confidence judgments. Both photographs and confidence scales were presented on-screen only until the time of response. After the completion of all trials, participants were administered a Deception Strategies Questionnaire to record whether participants tried to evade detection by employing specific eye movement strategies or manipulations of confidence.

#### Deception strategies questionnaire

A short questionnaire was designed to gauge whether participants attempted to adopt any behavioural strategies during the task. Participants were asked the following questions: Did you adopt any strategies during the task? (Participants circled a yes or no answer.); What strategies did you adopt when lying? What strategies did you adopt when telling the truth? What behaviours do you think are indicative of lying? What behaviours do you think are indicative of telling the truth? (Participants provided free text responses.) We also asked if participants thought they were successful in their attempt to conceal knowledge (circle yes or no and state why) and what photographs were hardest to lie about (newly learned or personally familiar). A final question asked participants whether they thought they displayed more of 12 listed behaviours depending on whether they were lying or telling the truth (cf. no difference), e.g. looked at the photograph more, looked away from the photograph, looked more at the confidence scale. No specific hypotheses were generated in relation to possible eye movement strategies.

## Data analysis

### Definition of interest areas

**Fig. 2:**
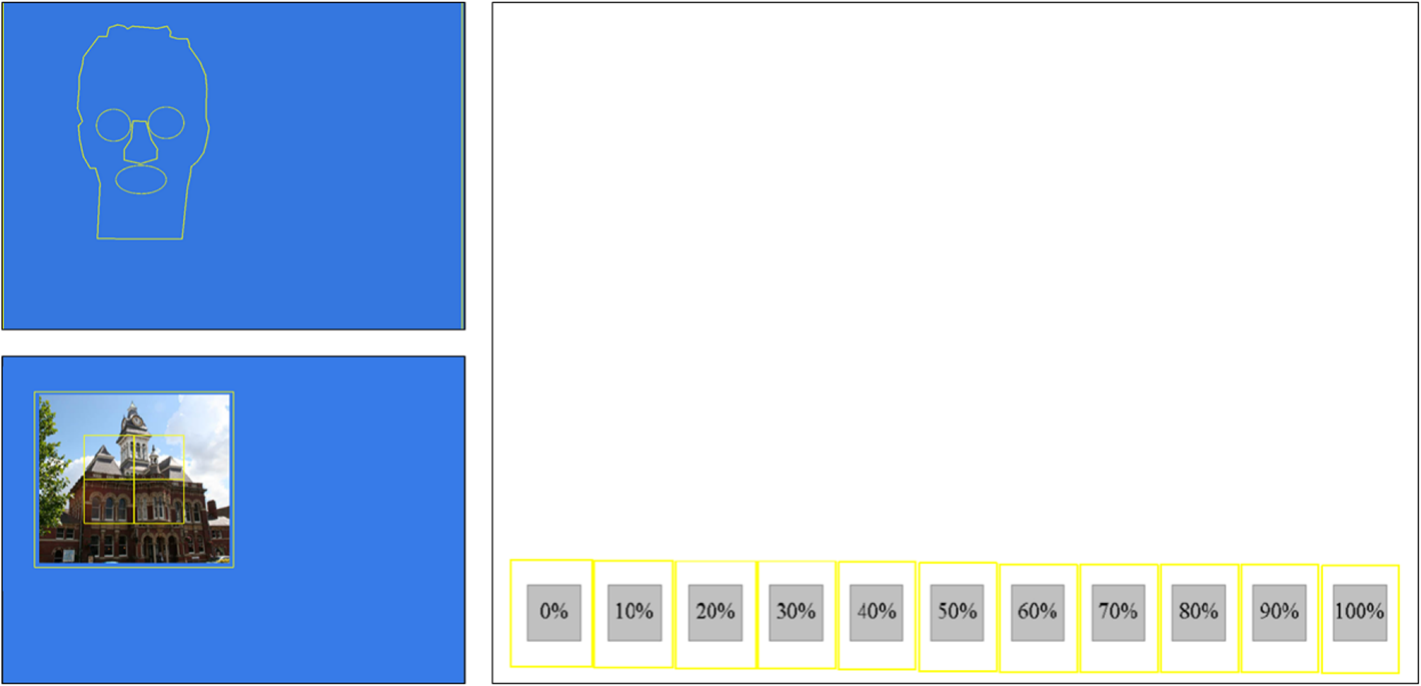
Interest areas defined: face (Experiment 1), non-face item (Experiment 2), confidence screen (Experiments1, 2) Photograph of actual person not shown as permissions not available

Figure 2 illustrates the defining of interest areas for one face (Experiment 1), one scene (Experiment 2) and the confidence screen (Experiments 1 and 2).

#### Experiment 1: faces

Consistent with previous research (e.g. Bate, Haslam, Tree, & Hodgson, 2008; Walker-Smith, Gale, & Findlay, 1977), five areas of interest were defined: right eye (left side of visual space), left eye (right side of visual space), nose, mouth and ‘outer’. The eyes, nose and mouth were grouped for analysis of the proportion of fixations made to the inner region of the face. The remainder of the image was classified as ‘outer’.

#### Experiment 2: non-face items

Using a method consistent with previous research on visual processing of scenes and objects (e.g. Smith & Squire, 2008), a fixed 4 × 4 grid was applied to each photograph to create 16 equally sized interest areas. The central four sections of the grid (top left, top right, bottom left, bottom right) formed the inner four inner regions of the photograph. The 12 remaining sections that surrounded the inner regions were grouped and merged to create one outer region. Each image therefore presented five interest areas for analyses, consistent with the number and structure of interest areas created for faces in Experiment 1.

#### Experiments 1 and 2: verbal confidence

Judgment options ranged from 0 to 100% in multiples of 10, such that each participant selected one out of 11 possible confidence judgments for each trial (0%, 10%, 20%, 30%, 40%, 50%, 60%, 70%, 80%, 90%, 100%). The mean and spread of values was analysed by entering SDs as the unit of analysis in repeated measures analyses of variance (RM ANOVAs) (see also Leongómez et al., 2014).

#### Experiments 1 and 2: confidence screens

To analyse the sampling distribution of eye movements during confidence judgments, boxes were drawn around each confidence decision box to create 11 interest areas: 0%, 10%, 20%, 30%, 40%, 50%, 60%, 70%, 80%, 90%, 100%. This allowed calculation of the number of interest areas (IAs) fixated and return fixations to the same regions of interest (Return Fixations) during deliberation of each confidence rating.

### Markers of recognition: theoretical justification

Consistent with previous research (Althoff & Cohen, 1999; Bate et al., 2008; Walker-Smith et al., 1977), we selected five markers of recognition based on their theoretical significance. The number of fixations made to the item was recorded as a general marker of cognitive effort for recognition (Num. Fixations). The number of different interest areas of the image viewed (IAs Visited) reflect the amount of information required to make a response. The number of return fixations to previously viewed areas of interest (two consecutive fixations in the same interest area belong to the same run and do not define a return) reflect the degree of uncertainty, or need to resolve ambiguity, in the decision-making process (Return Fixations; e.g. Barton, Radcliffe, Cherkasova, Edelman, & Intriligator, 2006). The proportion of fixations made to the inner regions of the image (Proportion Inner) reflects the extent to which recognition is achieved from inspecting critical features. Finally, average fixation duration (Ave Fix Duration) was recorded as an index of depth of processing. Average fixation durations are known to be modulated by recognition-orienting effects as well as cognitive load during lies (e.g. response conflict and strategies to conceal knowledge; Cook et al., 2012). Ave Fix Duration was calculated by summing the length of all fixations made to the item and then dividing by the number of total fixations. In sum, we predicted that recognition of familiar items would require less cognitive effort and that this would be reflected in fewer fixations, fewer IAs visited, fewer return fixations, fewer proportion of fixations directed to the inner regions of the image (faces, scenes and objects only) and longer fixation durations. Confidence judgments (0–100%) were recorded after each recognition response to assess the potential of patterns of confidence judgments as a novel marker of recognition.

#### Cohen’s *d* effect size analyses

The detection of concealed knowledge is primarily concerned with identifying robust markers of recognition via large effect sizes with narrow confidence intervals. Accordingly, we present our data across both experiments as Cohen’s *d* effect size differences with 95% confidence intervals (CIs). Here, Cohen’s *d* is calculated based on standardised difference scores for each dependent variable (e.g. Ben-Shakhar, 1985).

For each participant, half of the valid irrelevant items (after exclusions) were removed from each block to allow simulation of a virtual ‘innocent’ group of unknowledgeable responses for the receiver operating characteristic (ROC) analyses outlined below. The mean and *SD* of the remaining irrelevant responses were then used to compute z-scores separately for (1) the simulated ‘unknowledgeable’ innocent group and (2) the probe items for the knowledgeable group, compared to the same remaining irrelevants. The number of probe items used for the resampling was defined by valid irrelevants/2. Where the number of valid probe trials was less than irrelevants/2, the participant was dropped from analyses. The resampling process was repeated 1000 times to compute mean values for Cohen’s *d* and the ROC area under the curve (AUC), with 95% CIs. For participants who had the same score for every irrelevant item, resulting in a standard deviation of zero, z-scores were computed using the *SD* of the whole set of responses for that participant. Note that this is conservative since, if the response to probe items does differ from that of the irrelevant items, the *SD* of the whole set of responses will always be bigger than the *SD* of the irrelevant items alone, resulting in lower z-scores.

#### Receiver operating characteristic curves

For applied purposes, it is important to determine the efficacy of our fixation markers for distinguishing concealed recognition from correct rejection of genuinely unknown items. ROC curves plot the true positive rate (i.e. sensitivity) against the false positive rate and calculate an AUC. AUC values range from 0 to 1, with 0.5 indicating chance detection (see also Ben-Shakhar, Lieblich, & Kugelmass, 1970). To perform ROC classifications, average z-scores were calculated separately for the simulated unknowledgeable innocent group and probe items for the knowledgeable group, for each participant. MATLAB® was used to calculate Cohen’s *d* and AUCs with 95% bootstrapped CIs.

## Results

Results are presented here for the two main experimental blocks. Data from the practice block are reported in Supplementary Material Figure S1 (Experiment 1) and Figure S2 (Experiment 2).

*Experiment 1 exclusions.* We analysed 27 out of 33 participants following exclusion of four participants due to technical errors, and two participants who failed to complete the task according to instructions. After exclusion of famous celebrity trials, we extracted data for 60 trials for each of the 27 participants (20 responses to personally familiar faces, 20 responses to newly learned faces and 20 responses to unfamiliar faces). Out of a total of 1620 trials, we removed 54 responses to participants’ own faces (one per participant per block), 43 errors according to task instructions and 15 trials for responses faster than 300 ms or slower than 5000 ms (eg. Greenwald, Nosek & Banaji 2003). The total number of trials analysed for the full trial period was 1508 out of a possible 1620. For analyses of the first 750 ms, we further removed 49 trials faster than 750 ms. For one participant this left too few trials for resampling. Removal of the participant resulted in loss of a further 10 trials. In total, 1449 out of 1508 were analysed for the first 750 ms.

*Experiment 2 exclusions.* Two participants were excluded from Experiment 2 due to technical issues, leaving 35 out of 37 participants for analyses. The 35 participants completed 60 trials each, totalling 2100 trials. Seventy-two trials were removed for errors according to task instructions as well as 31 trials that were faster than 300 ms or slower than 5000 ms. For the full trial period we analysed 1997 out of 2100 trials. For analysis of the first 750 ms, a further 39 trials faster than 750 ms were removed. This resulted in too few trials for resampling for one participant. The participant was excluded from analysis, resulting in the loss of 13 more trials. In total, 1945 out of 1997 trials were included in analysis of the first 750 ms.

Trial errors are defined as incorrect ‘familiar’ responses to unfamiliar items; ‘unfamiliar’ responses to familiar items during honest identification; and ‘familiar’ responses to familiar items during concealed recognition.

### Markers of recognition

**Fig. 3:**
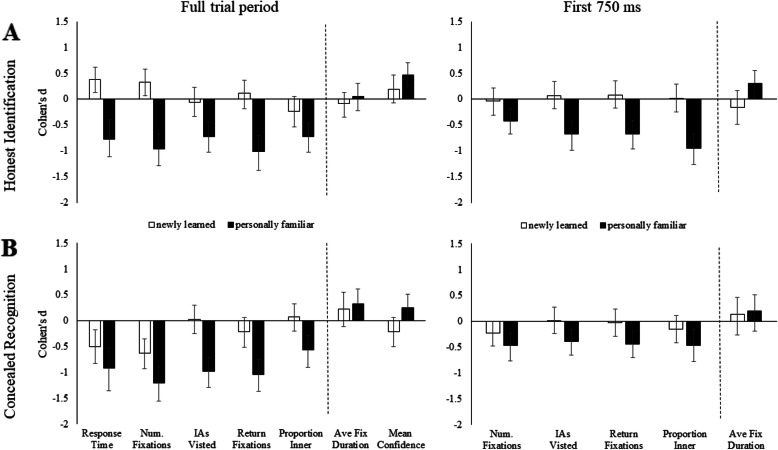
Cohen’s *d* effect size differences (95% CIs) for faces in Experiment 1**a** honest identification of familiar faces compared to unfamiliar faces, **b** concealed recognition of familiar faces compared to unfamiliar faces. On the *left*, data shown for the Full trial period are Response Times, Num. Fixations, IAs Visited, Return Fixations, Proportion Inner, Ave Fix Duration, Mean Confidence. On the *right*, we show re-analysis of the five fixation markers for the First 750 ms of the trial: Num. Fixations, IAs Visited, Return Fixations, Proportion Inner, Ave Fix Duration. Cohen’s *d* effect sizes are shown for comparisons between personally familiar faces and unfamiliar faces (*black bars*) and newly learned faces compared to unfamiliar faces (*white bars*). Tests of equality for effect sizes between honest and concealed conditions are presented in Supplementary Material Table S1

**Fig. 4:**
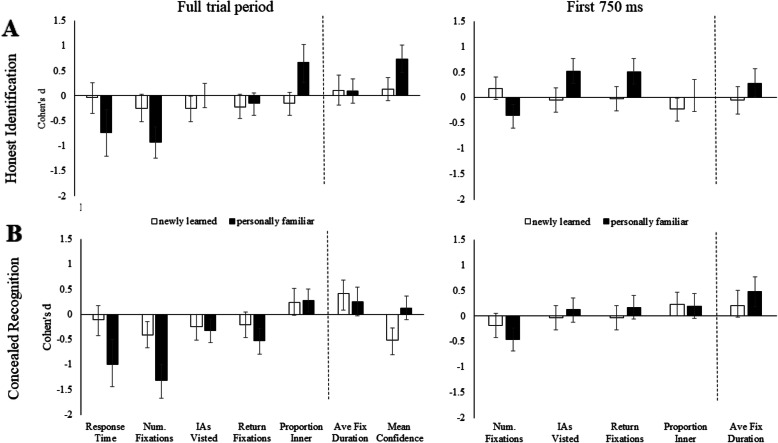
Cohen’s *d* effect size differences (95% CIs) for non-face items in Experiment 2**a** honest identification of familiar non-face items compared to unfamiliar non-face items, **b** concealed recognition of familiar non-face items compared to unfamiliar non-face items. On the *left*, data shown for the Full trial period are Response Times, Num. Fixations, IAs Visited, Return Fixations, Proportion Inner, Ave Fix Duration, Mean Confidence. On the *right*, we show re-analysis of the five fixation markers for the First 750 ms of the trial: Num. Fixations, IAs Visited, Return Fixations, Proportion Inner, Ave Fix Duration. Cohen’s *d* effect sizes are shown for comparisons between personally familiar non-face items and unfamiliar non-face items (*black bars*) and newly learned non-face items compared to unfamiliar non-face items (*white bars*). Tests of equality for effect sizes between honest and concealed conditions are presented in Supplementary Material Table S2

Figures 3 and 4 show Cohen’s *d* effect sizes (*M*_familiar_ – *M*_unfamiliar_) for faces in Experiment 1 and non-face items in Experiment 2. In each figure we show Cohen’s *d* for (a) honest identification and (b) concealed recognition trials. For calculation of Cohen’s *d*, familiar items were compared to unfamiliar items from the same block. Data shown for the *Full trial period* are Response Time, Num. Fixations, IAs Visited, Return Fixations, Proportion Inner, Ave. Fixation Duration, Mean Confidence judgments. Response times are included to show the relationship between fixation measures and response times. Negative values on the *y* axis (Cohen’s *d*) are consistent with faster response times, fewer fixations, fewer interest areas visited, fewer return fixations and a lower proportion of fixations to inner regions during recognition. Positive values on the *y* axis reflect longer fixation durations and higher mean confidence ratings during recognition. The vertical dashed line indicates the point in the figure at which data consistent with predictions should change from negative to positive values. Data presented for the *First 750 ms* are Num. Fixations, IAs Visited, Return Fixations, Proportion Inner, Ave Fix Duration. An exploratory analysis of the first 750 ms was conducted to investigate if markers of recognition were identifiable in less than a second. Establishing early markers of recognition may be useful if someone attempts to conceal recognition by responding quickly to all items. We selected the first 750 ms based on the emergence of early markers of recognition in previous research (Althoff & Cohen, 1999; Millen & Hancock, 2019; Ryan et al., 2007) and to minimise trial loss.

*Results from Experiment 1* (faces) are shown in Fig. 3. Recognition of personally familiar faces (black bars) was clearly differentiated from correct rejection of unfamiliar faces in multiple fixation measures and confidence ratings, across honest (a) and concealed recognition trials (b), with markers of recognition observed in the first 750 ms. Recognition of newly learned faces (white bars) was not consistently distinguished from unfamiliar faces across honest and concealed recognition trials.

*Results from Experiment 2* (non-face items) are shown in Fig. 4. Recognition of personally familiar non-face items (black bars) was identified by faster response times and fewer fixations during both honest (a) and concealed recognition trials (b). Fewer fixations were also observed in the first 750 ms of the trial. Recognition of newly learned non-face items (white bars) was not reliably detected across honest and concealed recognition trials. Detailed summaries are reported in the following sections.

### Experiment 1 (faces)

Figure 3 shows that recognition of personally familiar faces (black bars) was faster than correct rejection of unfamiliar faces during both honest (a) and concealed recognition (b). Consistent with predictions, fast recognition of personally familiar faces was differentiated by fewer fixations, fewer interest areas visited, fewer return fixations and a lower proportion of fixations to the inner regions of the face. The same pattern of fixation results was observed during honest identification (a) and concealed recognition trials (b) and during the full trial period and the first 750 ms of the trial. Longer fixation durations additionally indicated recognition in the full trial period of concealed recognition trials, but confidence intervals displayed substantial variability. Higher mean verbal confidence ratings were reported during recognition of personally familiar faces, compared to unfamiliar faces, in both honest and concealed recognition trials. Higher mean verbal confidence ratings during concealed recognition were also associated with more homogeneous (less variable) confidence ratings, Cohen’s *d* = −.52 [−.92, −.11]. Tests for equality of effect sizes between honest and concealed conditions revealed that the proportion of fixations to inner face regions in concealed trials was smaller, but still significant, than for honest trials. There were no other effect size differences between conditions (see Supplementary Material Table S1).

Figure 3a shows that, opposite to predictions, honest recognition of newly learned faces (white bars) was slower and required more fixations than correct rejection of unfamiliar faces. Conversely, Fig. 3b shows that concealed recognition of newly learned faces was faster and required fewer fixations than unfamiliar faces, which was consistent with our predictions. Accordingly, comparison of Cohen’s *d* across honest and concealed conditions confirmed differences in the pattern of data between honest and concealed recognition trials. There were no other significant differences in fixation measures (IAs Visited, Return Fixations, Proportion Inner, Average Fixation Duration) or verbal confidence ratings (mean ratings of variability)

### Summary of experiment 2 (non-face items)

Figure 4 shows that, consistent with predictions, recognition of personally familiar non-face items (black bars) was faster than for correct rejection of unfamiliar faces. During honest identifications (a), faster recognition was differentiated by fewer fixations and higher mean verbal confidence ratings, which was consistent with our predictions. Mean verbal confidence ratings were also more homogeneous during honest recognition of personally familiar non-face items compared to correct rejection of unfamiliar non-face items, *d* = −.65 [− 1.0, −.28]. Contrary to predictions, and results in Experiment 1, a larger proportion of time was spent viewing the inner regions on personally familiar non-face items, compared to unfamiliar non-face items. There were also no differences detected in the number of interest areas visited, return fixations or average fixation durations during the full trial period (see Supplementary Material Table S2). However, in the first 750 ms, whilst participants made fewer fixations to personally familiar compared to unfamiliar non-face items, these fixations were more widely distributed (more interested areas viewed, more return fixations), which was the opposite pattern to that predicted and different to the data for the full trial period. The data suggest that during honest identification of personally familiar non-face items, fixations are more distributed in the first 750 ms but not for the full trial period, where the majority of time overall is spent viewing the inner regions of the item.

During concealed recognition of personally familiar non-face items (b), faster recognition was differentiated by fewer fixations, fewer interest areas visited and fewer return fixations, compared to unfamiliar faces. As in Fig. 4a, a larger proportion of fixations was directed to the inner regions of personally familiar non-face items compared to unfamiliar ones. There were no differences in average fixation durations or mean verbal confidence ratings. Across honest and concealed recognition trials, there was no difference in response times, the number of fixations made or the proportion of fixations to inner regions. However, the average fixation duration, the number of interest areas visited, the number of return fixations and mean verbal confidence ratings were different between honest and concealed recognition trials. Recognition of newly learned non-face items was not reliably detected across honest and concealed conditions. However, fewer fixations did distinguish concealed recognition of newly learned items for unfamiliar non-face items in the full trial analysis, but not in the first 750 ms. Reported mean confidence ratings were unexpectedly lower during concealed recognition of newly learned items compared to unfamiliar non-face items.

In sum, detection of recognition was robust for personally familiar faces across multiple markers of recognition (e.g. fewer fixations, fewer interest areas viewed, fewer return fixations), but not for newly learned faces. Fewer fixations was the most reliable marker of recognition across experiments. Recognition was detected by fewer fixations during honest and concealed recognition of personally familiar faces, during concealed recognition of newly learned faces (but not honest identifications), during honest and concealed recognition of personally familiar non-face items and during concealed recognition of newly learned non-face items (bot not honest identifications). Fewer fixations observed during recognition of personally familiar faces (Experiment 1) and non-face items (Experiment 2) were also observed in the first 750 ms. Verbal confidence ratings clearly distinguished honest identification of personally familiar faces and non-face items from unfamiliar ones, but the equivalent contrasts for concealed recognition trials were variable. Confidence was not a reliable indicator of recognition for newly learned faces or non-face items. There were no consistent differences in eye fixation variables during viewing of the verbal confidence screen. See Discussion for further comment.

### Deception strategies (questionnaire data)

In Experiment 1 (faces), 3 out of 27 participants reported trying to *look more* at the face during lie trials, 6 reported trying to *look less* and 18 simply reported trying to do the same.

In Experiment 2 (non-face items), 10 out of 35 participants reported trying to look less during concealed recognition, 7 reported trying to fixate only on the centre of the image, 15 reported simply trying to do the same and 3 reported no strategies at all. In sum, all participants reported strategies, but most of these were ambiguous and showed no insight into how to conceal knowledge in fixation patterns. No further analyses were performed on these data. The data suggest that variability in patterns of fixations across different conditions is a consequence of general task demands and strength of recognition rather than explicit fixation strategies.

### Receiver operating characteristic curves

Tables 1 and 2 show that calculated AUCs were consistent with meaningful effect sizes in the main results section (markers of recognition until response). Concealed recognition of personally familiar faces was consistently detected above chance across all variables. Concealed recognition was detected by fewer fixations for all stimuli classes (faces and non-face items) and all levels of familiarity (newly learned and personally familiar items). Fewer fixations were observed in the first 750 ms for personally familiar face and non-face items, but less reliably for newly learned items.

**Table 1:** Areas under the curve [95% CIs] calculated for all markers of recognition in Experiment 1 for the full trial period and first 750 ms of the trial. Numbers in bold indicate AUCs above chance (i.e. the lower bound limit CI exceeds .50)

Experiment 1 Measure	Full trial		First 750 ms	
	Newly learned	Personally familiar	Newly learned	Personally familiar
Response Times	**.66 [.57, .75]**	**.80 [.73, .87]**	NA	NA
Num. Fix	**.67 [.59, .75]**	**.83 [.76, .89]**	.57 [.50, .65]	**.64 [.57, .72]**
IAs Visited	.49 [.04, .57]	**.76 [.69, .84]**	.50 [.42, .57]	**.61 [.53, .68]**
Return Fixation	.57 [.48, .64]	**.79 [.71, .86]**	.50 [.42, .58]	**.62 [.54, .70]**
Prop. Inner	.49 [.41, .57]	**.68 [.60, .77]**	.55 [.47, .62]	**.65 [.57, .72]**
Ave Fix Duration	.56 [.47, .65]	**.59 [.51, .67]**	.54 [.45, .63]	.57 [.49, .67]
Confidence	.44 [.36, .52]	**.58 [.51, .65]**	NA	NA

**Table 2:** Areas under the curve [95% CIs] calculated for all markers of recognition in Experiment 2 for the full trial period and first 750 ms of the trial. Numbers in bold indicate AUCs above chance (i.e. the lower bound limit CI exceeds .50)

Experiment 2 Measure	Full trial		First 750 ms	
	Newly learned	Personally familiar	Newly learned	Personally familiar
Response Times	**.69 [.62, .74]**	**.81 [.75, .86]**	NA	NA
Num. Fix	**.77 [.72, .82]**	**.87 [.83, .91]**	.55 [.48, .62]	**.62 [.55, .68]**
IAs Visited	**.68 [.62, .74]**	**.55 [.50, .61]**	.52 [.45, .61]	.46 [.39, .53]
Return Fixation	**.71 [.65, .77]**	**.60 [.55, .66]**	.53 [.45, .60]	.45 [.38, .51]
Prop. Inner	.47 [.42, .53]	.28 [.22, .34]	.43 [.35, .50]	.44 [.37, .51]
Ave Fix Duration	**.60 [.55, .65]**	**.66 [.61, .71]**	.42 [.35, .49]	.34 [.27, .41]
Confidence	.44 [.38, .50]	.43 [.36, .50]	NA	NA

## Discussion

Our aim was to identify reliable markers of recognition in fixations and confidence judgments. We tested the robustness of multiple fixation measures for the detection of concealed recognition across different classes of photographic stimuli (faces and non-faces) and different types of familiarity (personally familiar and newly learned). For Experiment 1, we predicted that recognition of personally familiar faces would be observed robustly across multiple markers of recognition, compared to newly learned faces. In Experiment 2, we also predicted that recognition of personally familiar items would be more reliably detected, compared to newly learned familiarity. We further predicted that confidence judgments would be higher and more stable for all familiar items (faces and non-faces) compared to correct rejection of genuine unfamiliar items. Our key finding, that detection of recognition of personally familiar faces was robust whereas other conditions were substantially more variable, is discussed in detail below.

As predicted, recognition of personally familiar faces was reliably detected across multiple markers of recognition irrespective of honesty. Specifically, recognition of personally familiar faces was marked by faster response times, fewer fixations, fewer areas of the face viewed, fewer return fixations to previously viewed areas of the face, a smaller proportion of fixations to inner face regions and longer fixation durations, compared to unfamiliar faces. Robust differences observed in fixation patterns between familiar and unfamiliar faces across measures confirm that processing of well-known faces is distinct from that for unfamiliar faces (see Johnston & Edmonds, 2009 for a review), and that these differences in fixations can be used to detect concealed recognition of well-known faces (Lancry-Dayan et al., 2018; Millen et al., 2017; Millen & Hancock, 2019). The finding that the same pattern of results was observed during both honest and concealed recognition of personally familiar faces confirms the robustness of our recognition markers for detection of concealed recognition of personally familiar faces (e.g. Millen et al., 2017; Millen & Hancock, 2019). The emergence of early markers of familiar face recognition in the first 750 ms is consistent with previous research (e.g. Althoff & Cohen, 1999; Ryan et al., 2007) and signifies the potential for recognition detection if deceivers attempted to respond quickly to all faces. The exception that longer fixation durations indexed recognition exclusively during concealment (e.g. Millen & Hancock, 2019), but not honest identification, highlights the key role of response conflict in eliciting differences in fixation durations (e.g. Cook et al., 2012). Differences in mean verbal confidence judgments, which were higher and less variable for personally familiar faces compared to correct rejection of genuinely unfamiliar faces, provided converging evidence that personally familiar memories were represented differently in memory. In sum, liars were unable to conceal recognition of personally familiar faces. As such, eye tracking shows clear potential for uncovering networks of criminals who are associated or personally known to each other in real life.

By contrast, markers of recognition for newly learned faces were not consistent across honest and concealed recognition trials. Although faster responses and fewer fixations indexed concealed recognition of newly learned faces as predicted, slower response times and an increase in fixations were unexpectedly observed during honest identification of newly learned faces. Although slower response times (Suchotzki, Verschuere, Peth, Crombez & Gamer, 2015) and more fixations (Seymour, Baker, & Gaunt, 2005) are reported in reaction time-based CITs during denial of recognition, this pattern of responding is not typically observed during honest identifications. In fact, slower response times and increased fixations are classically only reported in the concealed knowledge literature when using a presentation ratio of one familiar item to many unfamiliar items and a concealment instruction, which is fundamental to eliciting response conflict that drives the slower responding. When participants were instructed to respond honestly to items presented using a 1:1 ratio, faster responses (Suchotzki et al., 2015) and fewer fixations (Seymour et al., 2005) were reported. This change in pattern of responding represents a shift in the theoretical premise of the test to one that is consistent with theoretical models of recognition, wherein familiar items are recalled from memory faster.

Because we did not present few familiar items among many unfamiliar items, we did not predict slower responding or an increase in fixations for either honest or concealed recognition trials. Our experiment was designed to tap into effects corresponding to different types of familiarity, and so we presented many familiar faces (75%) among few unfamiliar faces. Participants lied about one group of familiar faces per block, whilst telling the truth about all other faces, which means approximately half of all responses in each test block were ‘familiar’ judgments. Considering the presentation ratio of our stimuli and the memory load of the task, we expected that participants would have to carefully inspect each face before executing the appropriate response selection, and that items stored in memory would be recalled faster. Accordingly, we expected our pattern of data to replicate honest responding observed in standard recognition paradigms (e.g. Althoff & Cohen, 1999) as opposed to slower responding in reaction-time-based CITs (Suchotzki, Verschuere, Van Bockstaele, Ben-Shakhar, & Crombez, 2017).

Consequently, we consider that the most probable explanation for the delay in responding to newly learned faces was that participants found it more effortful to recall these newly learned faces whilst they were incentivised to focus on the concealment of personally familiar faces (see Krebs, Boehler, & Woldroff, 2010 on the influence of reward on conflict processing). If participants prioritised the concealment of personally familiar faces, then it is feasible that the process of recollection and response planning for the newly learned faces might be delayed under competing processing demands (Seymour, 2001; Walczyk, Harris, Duck, & Mulay, 2014). We know that newly familiar faces are distinctly effortful to recall and thus are more likely to be subject to changes in task demands when memory load is high (Hancock et al., 2000). The finding that reverse effects were observed during recognition of newly learned faces, but not during recognition of personally familiar faces in Experiment 1 (or newly learned or personally familiar non-face items in Experiment 2), highlights the unreliability of fixation markers for detecting recognition of newly learned faces. Our findings are consistent with eye movement research underlining the crucial role of cognitive factors such as degree of familiarity, explicit task instructions and stimulus presentation on attention during visual processing tasks (e.g. Loftus & Mackworth, 1978; Nahari, Lancry-Dayan, Ben-Shakhar, & Pertzov, 2019; Yarbus, 1967).

Markers of recognition for personally familiar non-face items in Experiment 2 were not as robust as those observed for faces in Experiment 1 (i.e. fewer markers of recognition were identified across honest and concealed trials). For example, during concealed recognition we observed multiple markers of recognition including faster response times, fewer fixations, fewer interest areas viewed, fewer return fixations to familiar compared to unfamiliar non-face items. The effects for response times and number of fixations were robust across honest and concealed recognition, but differences in the number of interest areas viewed and return fixations disappeared during honest identification of personally familiar non-face items. Fewer fixations indicated recognition in the full trial period and the first 750 ms of the trial. Conversely, none of the markers of recognition for newly learned non-face items were reliable across honest and concealed recognition trials. Fewer fixations and longer fixation durations indicated concealed recognition of newly learned faces, but not during honest recognition. Fewer fixations during concealed recognition were observed in the full trial period and the first 750 ms, but longer fixation durations were only present in the full trial period. Interestingly, fewer fixations indicated concealed knowledge of newly learned non-face items, but response times did not. It is possible that, in some conditions, number of fixations may be a more sensitive marker of recognition due to its higher temporal resolution. Reports of lower mean confidence ratings for familiar non-face items, compared to unfamiliar items, underline the lack of certainty for newly learned items in this condition. It is also interesting to note that participants spent most of their time viewing the inner region of familiar non-face items compared to unfamiliar ones. This finding was not what we expected, but it is partly consistent with previous research which reports a central viewing tendency for viewing of scenes in laboratory-based experiments, such that the focus of attention is allocated to the centre of the scene (Bindemann, Scheepers, Ferguson, & Burton, 2010; Tatler, 2007). Some researchers suggest that the centre of a scene provides an ideal location for efficiently extracting information as a whole (Renninger, Vergheese, & Coughlan, 2007).

Overall, the results of Experiment 2 further confirm that markers of recognition for personally familiar items are more reliable than for newly learned items. These findings are consistent with previous research by Peth et al. (2013), who found that recognition of crime details central to a mock crime was detected above chance, but details peripheral to the crime were not. We also observed that markers of recognition were fewer for personal familiar non-face items than for personally familiar faces, suggesting that personally familiar faces were more richly represented in memory. This is surprising, given that familiarity ratings (1 = not at all familiar, 7 = very familiar) for personally familiar non-face items in Experiment 2 were almost at ceiling (*M* = 6.59, *SD* = 1.05) compared to lower ratings for personally familiar faces in Experiment 1 (*M* = 4.90, *SD* = 0.82).

In short, liars were unable to conceal recognition of personally familiar faces, places and objects. Our findings support that recognition involving real-world personal familiarity is superior to newly learned familiarity based on a single image. In particular, multiple markers of recognition observed for personally familiar faces confirm that well-established familiarity is fast and relatively holistic, making detection by eye fixations straightforward. The current findings build on a few existing studies that emphasise the importance of encoding strength on fixations as markers of recognition (Millen et al., 2017; Millen & Hancock, 2019; Peth et al., 2013). Other variants of the CIT have also demonstrated that encoding strength interacts with detection by physiological measures (e.g. Carmel, Dayan, Naveh, Raveh, & Ben-Shakhar, 2003), response times (e.g. Georgiadou, Chronos, Verschuere, & Sauerland, 2019; Seymour & Fraynt, 2009) and event-related brain potentials (e.g. Meijer, Smulders, Merckelbach, & Wolf, 2007). The current study extends this research to show that newly learned faces or non-face items do not approximate personal familiarity. We propose that, when establishing the robustness of markers of recognition to detect intentional concealment, a clear distinction should be drawn between different types of familiarity (i.e. brief incidental exposure, newly learned faces, well-established personal familiarity), and that conclusions made when using one kind of familiarity should not be assumed to generalise to all types of familiar faces. This point is particularly pertinent for faces. Crucially, we demonstrate that suspects most likely to strongly deny their association (i.e. criminal gang members who know each other well) are the ones for whom it should be easiest to detect.

The current findings present a promising avenue for the use of eye fixations and memory confidence for concealed knowledge detection. However, we note three key points that warrant further investigation in future research. First, our exploration of verbal confidence ratings and fixation patterns during lies about recognition confidence showed that mean confidence ratings were higher and less variable for personally familiar faces (Experiment 1), but that there were no differences in the way that individuals looked at the confidence scale during recognition compared to deliberation of genuinely unfamiliar faces and non-face items. This exploration was a first step in evaluating a new approach to detecting lies about recognition drawing on reported meta-cognitive markers. However, some methodological issues may have limited the utility of our results. In the current experiment we chose to select an 11-point, 0–100% scale (cf. Sauer, Brewer, & Weber, 2008). However, we found that mean verbal confidence ratings were generally high, which may have contributed to ceiling effects and homogeneity of the confidence data. In addition, we observed that participants tended to look very little at the confidence screen during confidence decisions, thus generating no meaningful differences in fixation behaviour. Use of a scale that required more cognitive engagement, such as the movement of a slide on a continuous 0–100 scale or the use of verbally meaningful anchors on the scale, might have detected finer grained differences in confidence whilst also encouraging attention to the screen.

Debates regarding the best way to accurately assess the relationship between memory confidence and accuracy are complex, and the efficacy of numeric scales (verbal labels, for instance) for establishing a relationship between confidence and accuracy has been questioned (e.g. Weber, Brewer, & Margitich, 2008; Windschitl & Wells, 1996). Psychological research tends to support that individuals think about confidence in verbal terms. Accordingly, the American judicial systems suggest the use of verbal scales with witnesses. However, research findings on this topic are mixed. For example, Windschitl and Wells (1996) found that verbal measures of confidence were more sensitive to factors relating to uncertainty, whereas Weber et al. (2008) compared numeric confidence responses on an 11-point scale to verbal labels and found very little difference in the confidence-accuracy relationship. Identifying patterns of deceptive confidence reporting is important for the legal system, since confidence has such a strong impact on witness credibility. We suggest that future research should find ways to more actively engage deceptive individuals in measurable meta-cognitive processes during lies about confidence.

Second, in the current experiments, interest areas were manually drawn on our photographs. This method was selected because we wanted to establish simple markers of recognition that tapped into differences in fixation quantity and distribution that could be easily applied across different types of photographic stimuli. However, in the future we will explore more sophisticated methods for analysis that rely on participant-dependent hotspots, similar to the analysis of functional magnetic resonance imaging (fMRI) data (e.g. Cornelissen, Hancock, Kiviniemi, George, & Tovée, 2009; Lao, Miellet, Pernet, Sokhn, & Caldara, 2017). Saliency maps (Borji, Parks, & Itti, 2014; Borji & Itti, 2013) and meaning maps (Henderson & Hayes, 2018; Henderson, Hayes, Rehrig, & Ferreirs, 2018) will be useful to identify relevant parts of the image for more fine-grained analyses that address debates on what defines saliency (e.g. Hayes & Henderson, 2019).

Third, evaluation of concealment strategies in the current experiment revealed that most participants reported ‘trying to do the same’ during concealed trials, which had no apparent effect on detection of personally familiar face recognition. Accordingly, we can conclude that fixation markers for personal familiarity are robust in the context of ambiguous strategies, which is consistent with other concealed knowledge research using different methodologies (e.g. Ben-Shakhar & Elaad, 2003; Meijer et al., 2014). However, few studies have assessed the robustness of fixation measures during informed countermeasures to conceal recognition (Millen & Hancock, 2019; Peth et al., 2016; Nahari et al., 2019, this issue). Whereas Millen & Hancock (2019, this issue) found that fixation durations were largely robust to intentional eye movement strategies to look at every face the same way, Nahari et al. (2019) found that fixation duration as a marker of recognition in simultaneous displays of faces (one familiar face to many unfamiliar faces) vanished when participants were instructed to look at all faces equally. Alternatively, Peth and colleagues examined the effectiveness of physical and mental countermeasures (physical finger wiggling or mental imagining of emotional events) during recognition of incidentally encoded information, and found that fewer fixations were invulnerable to both countermeasures but that fixation durations were not. Substantially more research is required to establish the limits of fixation markers to detect concealed recognition under intentional countermeasures.

## Conclusions

The use of eye tracking for detecting recognition has clear potential for field use. Markers of recognition for faces, scenes and objects known in real life uncover intentional efforts to conceal knowledge. Fixation-based markers of recognition for personally familiar faces are particularly robust. Yet detection efficiency for less well encoded memories varies according to task demands such as stimulus class, familiarity and instructions to conceal. Further research is required to establish the robustness of different fixation markers during intentional countermeasures and to determine whether detection of less familiar faces, places and objects can be improved.

## Supplementary information

**Additional file 1. **Supplementary material

## Data Availability

The datasets generated and analysed during the current study are available at the Open Science Framework repository, https://osf.io/zm42f/?view_only=e5e7929760e64b029201dc0d010ac769.
