# Supplementary material for: Eye spy a liar: assessing the utility of eye fixations and confidence judgments for detecting concealed recognition of faces, scenes and objects
Source: Cogn Res Princ Implic. 2020 Aug 14;5:38. doi: 10.1186/s41235-020-00227-4 (PMC7427826; doi:10.1186/s41235-020-00227-4)
Supplement: Supplementary file 1 — Additional file 1. Supplementary material [file 41235_2020_227_MOESM1_ESM.docx]

Supplementary Material

**Figure S1.** Cohen’s d effect size differences (95% CIs) for Experiment 1 practice block: honest identification of familiar target faces compared to correct rejection of genuinely unfamiliar non-faces. In the practice block 30 trials were extracted for 27 participants (810 trials; 10 personally familiar, 10 newly learned and 10 unfamiliar for each participant). 745 out of 810 trials were analysed after removal of 27 participant responses to their own faces, 35 errors (9 errors to personally familiar faces, 13 errors to newly learned faces, 13 errors to unfamiliar faces, and 3 trials that were faster than 300ms or slower than 5000ms (1 unfamiliar, 2 newly learned).

**Figure S2.** Cohen’s d effect size differences (95% CIs) for Experiment 2 practice block: honest identification of familiar target non-faces compared to correct rejection of genuinely unfamiliar non-faces. In the practice block 30 trials were extracted for 35 participants (1050 trials; 10 personally familiar, 10 newly learned and 10 unfamiliar for each participant). 941 out of 1050 trials were analysed after removal of 73 errors (7 errors to newly learned non-face items, 66 errors to unfamiliar faces, and 36 trials that were faster than 300ms or slower than 5000ms (11 personally familiar, 14 newly learned, 11 unfamiliar).

Table S1. Results for tests of equality comparing the difference [95% CIs] between Cohen’s d effect sizes across (A) honest identifications and (B) concealed recognition in **Experiment 1**. Confidence intervals that intersect zero suggest no difference in mean effect sizes between the two conditions.

| **Experiment 1** Measure | **Full trial** | | **First 750 ms** | |
| --- | --- | --- | --- | --- |
|  | Newly learned | Personally Familiar | Newly learned | Personally Familiar |
| Response Times | .87 [.46, 1.3] | .23 [-.27, .80] | NA | NA |
| Num. Fix | .95 [.57, 1.3] | .24 [-.22, .68] | .02 [-.17, .56] | -.02 [-.36, .31] |
| IAs Visited | -.05 [-.37, .29] | .25 [-.13, .68] | .05 [-.31, .43] | -.29 [-.69, .07] |
| Return Fixation | .32 [-.08, .70] | .03 [-.45, .49] | .08 [-.30, .46] | -.23 [-.61, .13] |
| Prop. Inner | -.31 [-.71, .08] | -.18 [-.61, .27] | .18 [-.19, .56] | -.05 [-.91, -.07] |
| Ave Fix Duration | -.32 [-.73, .07] | -.28 [.-69, .06] | -.31 [-.78, .18] | .01 [-.35, .56] |
| Confidence | .37 [.01, .72] | .21 [-.11, .52] | NA | NA |

Table S2. Results for tests of equality comparing the difference [95% CIs] between Cohen’s d effect sizes across (a) honest identifications and (B) concealed recognition in **Experiment 2**. Confidence intervals that intersect zero suggest no difference in mean effect sizes between the two conditions.

| **Experiment 2** Measure | **Full trial** | | **First 750 ms** | |
| --- | --- | --- | --- | --- |
|  | Newly learned | Personally Familiar | Newly learned | Personally Familiar |
| Response Times | .07 [-.33, .49] | .28 [-.37, .94] | NA | NA |
| Num. Fix | .15 [-.21, .53] | .40 [-.02, .87] | .36 [.03, .07] | .11 [-.21, .43] |
| IAs Visited | .00 [-.33, .34] | .36 [.05, .70] | -.02 [-.36, .33] | .39 [.05, .74] |
| Return Fixation | -.01 [-.35, .34] | .38 [.04, .74] | .00 [-.33, .32] | .33 [.01, .64] |
| Prop. Inner | -.38 [-.74, -.03] | .37 [-.08, .80] | -.46 [-.08, -.14] | .36 [.01, .76] |
| Ave Fix Duration | -.31 [-.69, .11] | -.16 [-.52, .23] | -.28 [-.67, .11] | -.18 [-.59, .21] |
| Confidence | .64 [.09, .29] | .61 [.26, .96] | NA | NA |
